# Dictionary of Practical Medicine

**Published:** 1844-12

**Authors:** 


### Bibliographical Notices.

*A Dictionary of Practical Medicine:* comprising General Pathology, the Nature and Treatment of Diseases, Morbid Structures, &c., by James Copland, M. D., F. R. S., edited, with additions, by Charles A. Lee, M. D., part 1, New York, Henry G. Langley.

The above work needs no commendation at our hands; all who have seen it must admire it, for the extent and immense amount of the information it contains on every branch connected with the science of medicine. And the additions by Dr. Lee, consisting of the introduction of the American improvements, must greatly enhance its value in the eyes of American readers, as well as add to the stock of scientific medical knowledge to those of our transatlantic brethren, who can sufficiently lay aside their natural pride and prejudices, to view, with a calm and dispassionate eye, the labors and efforts of "Brother Jonathan" to heal the diseases, and relieve, as far as possible, the miseries and sufferings of mankind.

But there is another reason for calling special attention to this work, and that is its cheapness. In the language of the publisher, "the American edition, which will be comprised in twenty numbers, will punctually appear monthly; forming, when finished, three handsome octavo volumes, price $10, is one of the most valuable, as well as cheapest productions afforded to the medical profession."

